# Analysis and Synchronous Study of a Five-Dimensional Multistable Memristive Chaotic System with Bidirectional Offset Increments

**DOI:** 10.3390/e27050481

**Published:** 2025-04-29

**Authors:** Lina Ding, Mengtian Xuan

**Affiliations:** School of Electronics and Information Engineering, Heilongjiang University of Science and Technology, Harbin 150022, China

**Keywords:** bidirectional offset increment, transient attractor, homogeneous multistability, super-multistability, synchronous control

## Abstract

In order to further explore the complex dynamical behavior involved in super-multistability, a new five-dimensional memristive chaotic system was obtained by using a magnetically controlled memristor to construct a four-dimensional equation on the basis of a three-dimensional chaotic system, adding a five-dimensional equation and selecting parameter y as the control term. Firstly, the multistability of the system was analyzed by using a Lyapunov exponential diagram, a bifurcation diagram and a phase portrait; the experimental results show that the system has parameter-related periodic chaotic alternating characteristics, symmetric attractors and transient chaotic characteristics, and it also has the characteristics of homogeneous multistability, heterogeneous multistability and super-multistability, which depend on the initial memristive values. Secondly, two offset constants g and h were added to the linear state variables, which were used as controllers of the attractors in the z and w directions, respectively, and the influences of the bidirectional offset increments on the system were analyzed. The complexity of the system was analyzed; the higher the complexity of the system, the larger the values of the complexity, and the darker the colors of the spectrogram. The five-dimensional memristive chaotic system was simulated using Multisim to verify the feasibility of the new system. Finally, an adaptive synchronization controller was designed using the method of adaptive synchronization; then, synchronization of the drive system and the response system was realized by changing the positive gain constant k, which achieved encryption and decryption of sinusoidal signals based on chaotic synchronization.

## 1. Introduction

Chaos is a form of motion specific to nonlinear dynamical systems [1]. Since the 1990s, the study of chaos and related disciplines has been promoted and developed, and the requirements for chaos in different fields have also been developed. People have constructed many new chaotic systems based on existing chaotic systems, including the introduction of a memory resistor to construct a new chaotic system. Memristive components have unique nonlinear characteristics and are easy to combine with oscillator circuits to generate complex and variable chaotic signals; thus, they have attracted much attention in the design of chaotic circuits and other aspects. The memristor is the fourth basic circuit element that reflects the relationship between magnetic flux and charge [2], and it has the characteristics of fast operation, programmability, low energy consumption and nonlinearity. Another definition of chaos has been given based on homoclinic and heteroclinic cycles, which play an important role in the study of chaotic dynamics. The famous Shil’nikov theorems for differential vector fields showed that the existence of a homoclinic or a heteroclinic cycle implies the existence of a countable number of horseshoes in a neighborhood of this cycle under certain conditions [3]. Therefore, chaotic systems constructed by memristors are usually capable of generating complex dynamical behaviors, and they have a wide range of applications in the fields of neural networks [4,5,6], chaotic circuits [7] and image encryption [8,9].

At present, there are two main methods for constructing memristive chaotic systems: one method is to introduce the memristor into the original system as a feedback term or use the memristor to replace the coupling parameters of the original system; the other method is to utilize the memristor to replace the linear or nonlinear device of the original circuit. In 2014, Ontañón-García et al. proposed a chaotic multivolume attractor generation mechanism based on unstable dissipative systems, which changes the equilibrium position of the unstable dissipative systems by switching the control law to generate a multivolume chaotic attractor. They explained the different concepts involved, such as hysteresis and the step function, from a unified viewpoint, extended the concept of chaos from R3 to hyperchaotic multivolume systems in Rn, and also verified the validity of the theory using the example of a synchronous communication system [10]. In 2018, Chen et al. proposed a memristive circuit based on an active bandpass filter, which has super-multistability and rich dynamic behavior [11]. In the same year, Wang et al. established a corresponding Simulink simulation model based on the mathematical models of three memory elements: the memristor, the memristor container and the memristor inductor; then, they studied the effects of frequency and amplitude on the respective device models, which provided a theoretical basis for subsequent application research [12]. In 2020, Zeng et al. designed a bimemristive chaotic circuit without inductance, revealing unique multistability characteristics through numerical simulation and hardware implementation [13]. In 2020, Escalante-González et al. developed a class of equilibrium-free systems that exhibit convolutional attractors and vector fields that are differentiable, constructing higher-order hyperchaotic systems through a special coupling mechanism while maintaining equilibrium-free properties. Tools such as the Lyapunov exponential, the Kaplan–Yorke dimension and the Poincaré cross-section were used to numerically investigate the nine-dimensional system, demonstrating the potential and flexibility of the method in generating complex dynamical behaviors [14]. In 2021, Li et al. designed general mathematical models of a voltage-controlled memristor, a charge-controlled memory vessel and a magnetron mempanic, respectively. They derived a five-dimensional memory hyperchaotic system after simple parallel connection of the three designed memory elements, which employs a novel “walnut-shaped” hyperchaotic attractor with three positive Lyapunov exponents and exhibits dynamic behavior with regard to parameter symmetry [15]. In 2021, Caldarola et al. studied the chaotic behavior in Chua oscillators and Chua circuits with memristors using the super-trackline method. By varying the control parameters of the circuits, bifurcation maps were obtained, and the super-trackline functions associated with these bifurcation maps were computed by numerical integration using the Lunger–Kutta method. Their study demonstrates the effectiveness of the super-trackline method in analyzing memristive chaotic systems and provides a new perspective for understanding the dynamical behavior of such systems [16]. Therefore, this paper uses magnetically controlled memristors to construct high-dimensional chaotic systems, generating richer dynamic behaviors to solve the above problems.

In recent years, multistability has become an important research object in memristive chaotic systems [17,18]. Multistability and super-multistability have become an important research direction in chaos-related fields. Multistability means that when the system parameters are fixed, the system can present many different stable states under different initial conditions, such as point attractors, periodic states, quasi-periodic states and chaotic states. These different stable states are called coexisting attractors of the system. Super-multistability is the extreme case of multistability, where the system can present the phenomenon of an infinite number of coexisting attractors under fixed parameters due to a change in the initial conditions. This phenomenon is usually associated with some special structures or nonlinear properties of the system, such as the introduction of nonlinear components and memristors. Systems with super-multistability exhibit great richness and complexity in their dynamical behavior and are extremely sensitive to the initial conditions. Although there have been many reports on the multistability and super-multistability of memristive chaotic systems, most of the papers have studied the heterogeneous multistability of chaotic systems, and few have reported on homogeneous multistability. Heterogeneous multistability refers to the fact that a chaotic system has a variety of chaotic attractors with different structures under the same system parameters. Homogeneous multistability, on the other hand, refers to the fact that chaotic systems can produce attractors with different amplitudes and spatial positions but with the same structure [19].

In 2017, Zhou introduced a smooth magnetron memristor based on a pseudo-four-wing chaotic system and transformed it into a wing-variable chaotic system, which could generate two-wing, three-wing and four-wing chaotic attractors as well as four-wing hyperchaotic attractors and coexisting two-wing attractors [20]. In 2019, Atefeh et al. proposed a five-dimensional chaotic system with super-multistability, which had a curvilinear line equilibrium point that could generate an infinite number of coexisting attractors with super-multistability [21]. In the same year, Yuan proposed a three-order memristive chaotic system with a minimal structure, in which the hidden attractors exhibited homogeneous multistability, heterogeneous multistability and super-multistability [22]. In 2020, Kengne et al. constructed a series of asymmetric chaotic circuits and revealed a variety of asymmetric coexistence bifurcation behaviors induced by asymmetric circuits through numerical simulations and hardware circuit implementation; they observed a variety of different types of asymmetric coexistence attractors [23]. In 2021, Gu et al. proposed a fractional-order conservative chaotic system with no equilibrium points, and the theoretical analysis and numerical simulations revealed the hidden super-multistable phenomenon caused by the initial shifts in three dynamical characteristics, including chaos, quasi-periodic and period [24].

Chaotic synchronization refers to the process by which two or more chaotic systems are controlled by internal coupling or external inputs so that the system motion state eventually reaches consistent dynamic states under different initial states. Chaos synchronization is an important part of chaos research, from theory to practical application, and the theory of synchronization has been widely employed in practical engineering fields such as communications, laser networks, chemical reactions, biological systems, mechanical systems and robotics. Therefore, the exploration of the investigating of chaotic synchronization has become an important topic in the study of chaos. In 2018, Zheng et al. extended the finite-time theory of fractional-order systems to hidden attractor chaotic systems, successfully realizing the self-synchronous control of the system [25]. In 2020, Rashidnejad et al. studied the synchronization problem of chaotic systems with uncertain parameters by using finite-time fractional adaptive sliding-mode control technology [26]. In 2021, Zheng et al. used Lyapunov’s stability theory and adaptive control method to study synchronization between uncertain chaotic systems of high-dimensionally mismatched systems [27]. In 2022, Qu et al. constructed a chaotic synchronization system and a secure communication model based on the synchronous control theory and realized experiments of memristive chaotic synchronization and chaotic encryption/decryption circuits through hardware experiments; the experimental results confirmed that the circuit had the advantages of high reliability and robustness [28].

Chaos is used for information encryption in two primary approaches: The first involves confidential communication based on chaotic modulation. The second involves cryptographic applications based on chaotic mapping. Chaotic secure communication techniques can be categorized into three groups based on different modulation methods: chaos masking [29], chaos modulation [30] and chaos shift keying [31]. Liu et al. implemented the projection synchronization of the two systems and further designed a new chaotic communication scheme, which encrypts the message by means of chaos masking. The sending signal is the derivative of the product of the plaintext message and the chaotic signal, which contains an arbitrary complex calibration function and makes the sending signal possess higher complexity [32]. In 2020, Khan et al. investigated a new 4D fractional-order chaotic system by constructing nonlinear control inputs to achieve staggered-phase synchronization and inverse-phase synchronization with its parallel system; based on this, the staggered-phase synchronization in the parallel system was exploited to mask the encryption of the plaintext analog sequences using the three-dimensional sequences [33]. So, this paper is built upon the realization of chaotic synchronization; the encryption and decryption of sinusoidal signals are realized by the methods of chaotic masking.

Therefore, a five-dimensional memristive chaotic system with homogeneous multistability, heterogeneous multistability and super-multistability coexistence is constructed in this paper. Firstly, Lyapunov exponential diagrams, bifurcation diagrams and phase portraits are used, and the phenomenon of super-multistability controlled by parameter and initial value variation is analyzed. Secondly, the bidirectional offset increment is investigated by introducing two offset constants, and the complexity of the system is also analyzed; the experimental results show that the system has a high complexity, and the feasibility of the system is verified by building a simulation circuit using Multisim. Finally, through the method of adaptive synchronization, an adaptive synchronization controller is designed, the drive system and the response system are synchronized and the application of secure communications is realized.

The rest of the paper is organized as follows. Section 2 constructs a five-dimensional chaotic system based on a magnetically controlled memristor; the system is verified as a chaotic system by a combination of theory and experiments, and the basic dynamic characteristics of the chaotic system are analyzed. Section 3 explores the influence of system parameters and memristive parameters on the dynamics of chaotic systems and explores the influence of the initial value of the memristor on the transient attractors. These investigations demonstrate that the system parameters have strong sensitivity and can be better applied to image encryption or secure communication. Section 4 uses the Multisim circuit implementation to simulate the chaotic system, and the phase portraits of the system are observed through a simulated oscilloscope, which verifies the correctness of the chaotic system. Section 5 uses the adaptive control method to realize the synchronous control of two chaotic systems with the same structure, and the application of chaotic system synchronization in secure communication is realized. Section 6 summarizes the work of this paper and provides a future outlook.

## 2. Design of Memristive Chaotic System

### 2.1. Model Construction of Chaotic System

In 2008, Yang, Chen, and colleagues proposed a typical three-dimensional chaotic system [34] featuring a saddle point and two stable foci, which was mathematically modeled as per Equation (1):

(1) $$\left\{\begin{array}{l} \overset{•}{x}=-ax+ay\\ \overset{•}{y}=bx-xz\\ \overset{•}{z}=xy-cz \end{array}\right..$$

where x, y and z are the system state variables, and a, b and c are all positive parameters.

In this paper, a magnetically controlled memristor [35] is used; the magnetically controlled memristor can be represented by a characteristic curve q=q(φ) through the origin in the φ–q plane. If a magnetically controlled memristor element is defined to have a smooth, nonlinear characteristic curve $q(\varphi )=a\varphi +b\varphi^{3}$ that monotonically increases three times, which yields its memristor function W(φ), the mathematical model is then shown in the following equation:

(2) $$W(\varphi )=\frac{dq(\varphi )}{d\varphi }=m+n\varphi^{2}.$$

The volt–ampere characteristic between the current flowing through and the voltage across the terminals can be described as i=W(φ)v and $\overset{•}{\varphi }=v$, where i and v denote the terminal current and terminal voltage of the memristor, respectively, and W(φ) is a nonlinear function that depends on the memristor’s internal state variable q, where m>0, n>0.

The memristive function W(u) of the magnetically controlled memristor is introduced into the fourth equation of system (3). The control voltage y is selected as the fifth equation, and a linear term w is added; all memristive terms in system (1), including the xz term in the second equation of system (1) and the xy term in the third equation, are multiplied by z, a linear term w is added to the first equation of system (1) and a constant term 1 is added to the second equation. By further introducing the five-dimensional equation, a new five-dimensional memristive chaotic system is finally obtained. The mathematical model of the new five-dimensional memristive system is presented in the following equation:

(3) $$\left\{\begin{array}{l} \overset{•}{x}=-ax+by+w\\ \overset{•}{y}=cx-xz^{2}+1\\ \overset{•}{z}=xyz-dz\\ \overset{•}{w}=kW(u)y-w\\ \overset{•}{u}=y \end{array}\right..$$

In the above, x, y, z, w and u are the five state variables of system (3), k is the coupling strength of the memristor and a, b c and d are the control parameters of the system.

### 2.2. Chaotic System Validation

#### 2.2.1. Phase Portraits

The Lyapunov exponent describes the exponential divergence rate of perturbed initial conditions and serves as an effective tool for chaos identification. With fixed parameters a=25, b=30, c=25, d=10, k=20, m=1 and n=0.01 and with the initial conditions (1, 1, 1, 1, 1) unchanged, the Lyapunov exponents are $LE_{1}=3.7264$, $LE_{2}=0.001$, $LE_{3}=-0.3222$, $LE_{4}=-1.7298$ and $LE_{5}=-27.9366$, and the Lyapunov exponent dimension of the system is given by the following equation:

(4) $$D_{L}=j+\frac{1}{\left|LE_{j+1}\right|}\sum_{i+1}^{j}LE_{i}=4+\frac{LE_{1}+LE_{2}+LE_{3}+LE_{4}}{\left|LE_{5}\right|}=4.05997.$$

**Figure 1:**
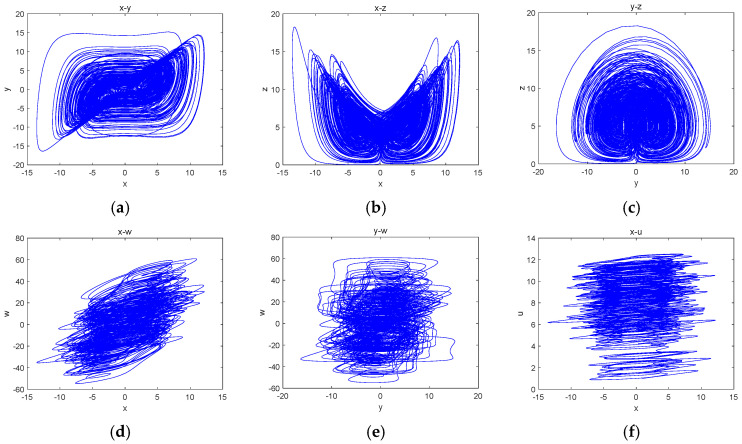
Projections of the attractors on different planes: (**a**) the x-y plane; (**b**) the x-z plane; (**c**) the y-z plane; (**d**) the x-w plane; (**e**) the z-w plane and (**f**) the x-u plane.

The projections of the attractors on different planes were plotted through MATLAB software 24.2 simulations, as shown in Figure 1.

#### 2.2.2. Time-Domain Waveforms and Poincaré Cross-Section

The time-domain waveform exhibits an irregular noise-like waveform, which can demonstrate a typical state of chaotic phenomena in Poincaré theory, and the trajectories of continuous motion can be effectively analyzed by a specific cross-section: the Poincaré cross-section. This cross-section allows the trajectory intersections across the cross-section to represent the motion characteristics in a concise and intuitive way.

**Figure 2:**
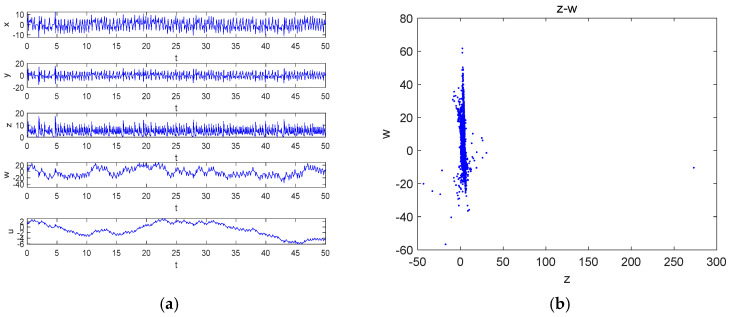
Time-domain waveforms and Poincaré cross-section with parameters a=25, b=30, c=25 and d=10: (**a**) the time series of x, y, z, w and u; (**b**) Poincaré cross-section on the plane z-w.

Keeping the parameters and initial values unchanged, the time series of x, y, z, w and u are shown in Figure 2a, and the Poincaré map on the z-w plane is presented in Figure 2b.

#### 2.2.3. The 0–1 Test

The states of complex chaotic systems are similar and difficult to distinguish during the transition process. It is difficult to distinguish these similar behaviors by using Lyapunov exponential diagrams and the corresponding bifurcation diagram, so in order to analyze the dynamical behaviors of the system more accurately, we introduce another test method, the 0–1 test. The test is used to determine the state of a system by analyzing the observable dataset associated with the dynamical behaviors of the system. The 0–1 test determines the state of the system by analyzing an observable dataset associated with the system’s dynamics. By constructing a stochastic dynamic process, the scale of the stochastic process changes over time can then be investigated. The 0–1 test provides a simple and intuitive test for the trajectory of the system in the (p, s) plane, regardless of whether the dynamical system is chaotic. The 0–1 test determines the state of a system by analyzing an observable dataset associated with the system’s dynamical behavior. Given an observation φ(i) for i=1,…, N and c∈(0,π), the p(n) and s(n) formulas are presented as follows.

(5) $$p(n)=\sum_{i=1}^{n}\varphi (i)\cos(ic),n=1,2,\ldots ,N$$

(6) $$s(n)=\sum_{i=1}^{n}\varphi (i)\sin(ic),n=1,2,\ldots ,N$$

where φ(i) is the observable dataset, and p and s are two time-dependent variables.

Based on the functions p(n) and s(n), the mean square displacement is defined as follows.

(7) $$M(n)=\underset{N\to \infty }{\lim}\frac{1}{N}\sum_{i}^{N}{\left[p_{c}(i+n)-p_{c}(i)\right]}^{2}+{\left[s_{c}(i+n)-s_{c}(i)\right]}^{2}$$

The expectation E(φ) is given as follows.

(8) $$E(\varphi )=\underset{N\to \infty }{\lim}\frac{1}{N}\sum_{i=1}^{N}\varphi (i)$$

**Figure 3:**
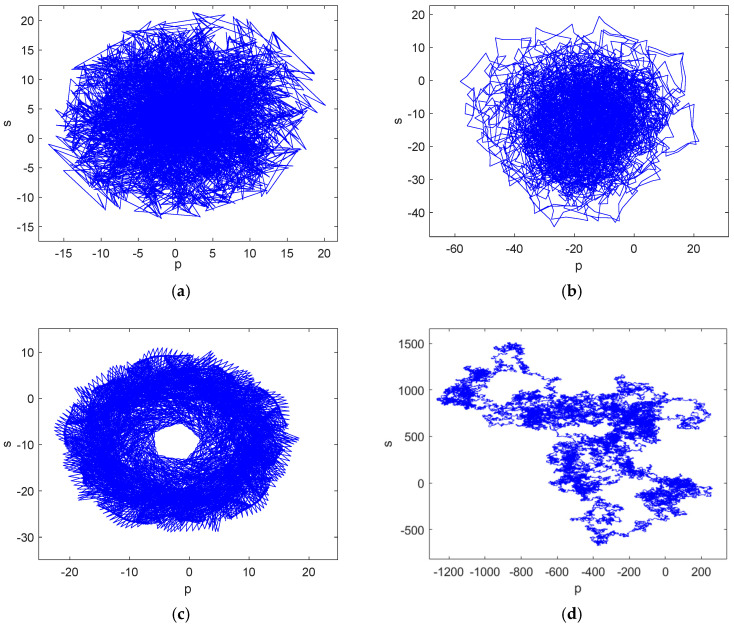
The 0–1 test varying with a: (**a**) a=12.7; (**b**) a=14.2; (**c**) a=16.5; (**d**) a=25.

If the trajectories of the functions p(n) and s(n) exhibit irregular Brownian motion, the system is in a chaotic state; if the trajectories of p(n) and s(n) are bounded, the system is in a periodic state [36]. The 0–1 test method is adopted to analyze its chaotic properties, and the 0–1 test results obtained by taking a=12.7, 14.2, 16.5 and 25 are presented in Figure 3.

From the figure above, it can be seen that when the parameter a varied, the results of the 0–1 test were different and the two cases of regular bounded circles and irregular unbounded growth appeared, and the specific results were analyzed, as shown in Table 1.

### 2.3. System Dynamics Analysis

#### 2.3.1. Symmetry and Dissipation

If a chaotic system has symmetry, then it is likely that its attractors appear in pairs and also have symmetry. If the coordinate transformation (x, y, z, w, u)→ (−x, −y, z, −w, −u) is performed, the memristive chaotic system (3) remains invariant, which indicates that system (3) is symmetric about the z-axis in state space.

The dissipative nature of system (3) can be characterized by its divergence, which is calculated as shown in Equation (9).

(9) $$\nabla v=\frac{\overset{•}{\partial (x)}}{\partial (x)}+\frac{\overset{•}{\partial (y)}}{\partial (y)}+\frac{\overset{•}{\partial (z)}}{\partial (z)}+\frac{\overset{•}{\partial (w)}}{\partial (w)}+\frac{\overset{•}{\partial (u)}}{\partial (u)}=-a+xy-d-1$$

The dissipative nature of the system depends on the sign of ∇v: when xy<a+b+1, ∇v<0, the chaotic system is dissipative; when xy>a+b+1, ∇v>0, the chaotic system is expansive; when xy=a+b+1, ∇v=0, the chaotic system is conservative.

When a=25 and d=10, ∇v=−36+xy. When xy<36, regardless of the values the other parameters of the system take, they do not affect the value of the dissipation degree, indicating that system (3) is dissipative, which suggests that the volume of the chaotic attractors in the phase space will be exponentially compressed to zero at an exponential $dv/dt=e^{-36+\mathrm{xy}}$ rate. When xy>36, system (3) is conservative; eventually, its trajectory will no longer be confined to the attractors.

#### 2.3.2. Equilibrium Point and Stability

To calculate the equilibrium point of the new system, let $\overset{•}{x}=\overset{•}{y}=\overset{•}{z}=\overset{•}{w}=\overset{•}{u}=0$; Equation (10) can be obtained as follows:

(10) $$\left\{\begin{array}{l} -ax+by+w=0\\ cx-xz^{2}+1=0\\ xyz-dz=0\\ kW(u)y-w=0\\ y=0 \end{array}\right..$$

By substituting the fifth equation y=0 into the third and fourth equations, w=0, z=0 and the first equation x=0, but the second equation x≠0. So, from the first equation and the second equation, it can be seen that a contradiction arises between the two equations, and Equation (13) has no solution, so system (3) has no equilibrium points. So, the system does not have equilibrium points, but it can produce chaotic behavior, its trajectory is complex, with complex folding, and it has a repetitive and extended structure, but it is bounded.

#### 2.3.3. Bidirectional Offset Incremental Control

Offset increment control is a chaotic attractor displacement phenomenon [37]. By adjusting the magnitude of the offset constants, the spatial position of the attractors is changed, and it is ensured that the topology of the attractors before and after the displacement remains basically the same, thus realizing the conversion between bipolar signals and unipolar signals in order to meet the needs of chaotic signals in different application fields. Two offset constants g and h are introduced into Equation (3), which are used as the controllers of the attractors in the z direction and w direction, respectively, and the modified system equation is as follows:

(11) $$\left\{\begin{array}{l} \overset{•}{x}=-ax+by+(w-h)\\ \overset{•}{y}=cx-x{\left(z+g\right)}^{2}+1\\ \overset{•}{z}=xy(z+g)-d(z+g)\\ \overset{•}{w}=k(m+nu^{2})y-(w-h)\\ \overset{•}{u}=y \end{array}\right..$$

**Figure 4:**
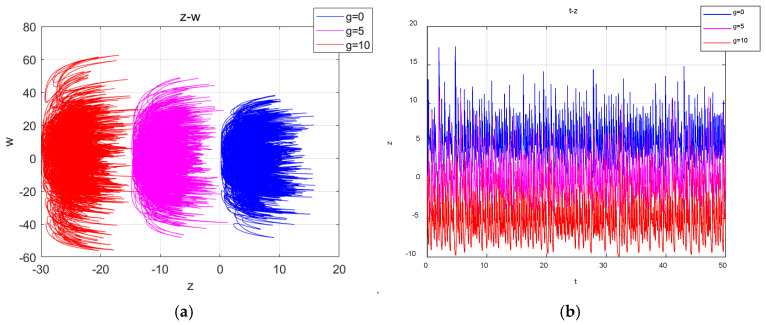
Projection of the attractors and time series in the z-w plane for different values of g: (**a**) the projection of the attractors on the plane z-w; (**b**) the time series of z.

With the system parameters a=25, b=30, c=25, d=10, k=20, m=1 and n=0.01 fixed and the initial conditions (1, 1, 1, 1, 1) unchanged, when the value of the offset constant g takes values of 0, 5 and 10, the corresponding attractors are shown in Figure 4a, while the time-domain waveforms are shown in Figure 4b. It can be observed that as the value of g increase, the system trajectory shifts uniformly from the positive axis of z to the negative axis of the z direction, thereby achieving the polarity reversal.

**Figure 5:**
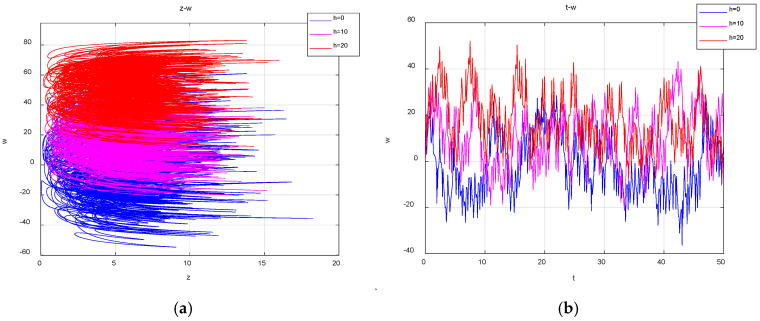
Projections of the attractors and time series in the z-w plane for different values of h: (**a**) the projection of the attractors on the plane z-w; (**b**) the time series of w.

Keeping the other parameters unchanged, when g=0, the initial value is (1, 1, 1, 1, 1); when the values of the offset constant h are 0, 10 and 20 respectively, the corresponding attractors are shown in Figure 5a, and the time-domain waveform is shown in Figure 5b. As can be observed, the motion trajectory of the system moves uniformly in the positive direction of z as the value of h increases. In summary, the dynamical behavior of the system is always kept in a relatively stable range during the change in the offset constants g and h. While the positions of the attractors are changed, not only is the chaotic property maintained, but the conversion of the signal polarity is also realized, which suggests promising applications for chaotic signals in the future.

## 3. Multistability Analysis of Memristive Chaotic System

In this section, the stability of a chaotic system is analyzed using the Lyapunov exponent, and the dynamic behavior of the system is visualized through a bifurcation diagram. The Lyapunov exponent is a key parameter that accurately quantifies the average exponential divergence of adjacent trajectories in the phase space of a chaotic system, and it serves as a key indicator to evaluate the sensitivity of the system to the initial conditions [38]. Determining the state of chaos by the Lyapunov exponential method is a form of quantitative analysis.

The physical meaning of the Lyapunov exponent can be understood by assuming that the initial value is taken as a particularly small R-dimensional sphere, which eventually changes to an ellipsoid as the dynamical behavior changes. In this process, all principal axes of the ellipsoid are ranked from the fastest to slowest rate of change, and the *i*th Lyapunov exponent is defined according to the *i*th principal axis rate of change.

(12) $$\sigma =\underset{n\to \infty }{\lim}\frac{1}{n}[\frac{p_{i}(n)}{p_{0}(n)}],i=1,2,3,\ldots ,R$$

When analyzing the dynamic behavior of chaotic systems, the state of the system can be evaluated by ranking the system’s Lyapunov exponents from high to low. The sign of the Lyapunov exponent characterizes the stability of the system. When the Lyapunov exponent is negative, the system is in a periodic state and has stable dynamic behavior. Conversely, when the Lyapunov exponent is positive, the system exhibits chaotic properties, and the system exhibits an unstable state. When the Lyapunov exponent is exactly zero, the system is on the boundary between stability and instability in a critical state.

A bifurcation diagram describes the state of a system as a certain system parameter or initial value changes. By looking at a bifurcation diagram, it is possible to determine whether a system is in a chaotic state or some other state, revealing the effect of a parameter or initial value on the state of the system.

Therefore, by fixing the other parameters, the data variations in the Lyapunov exponent spectrum and bifurcation diagram in different ranges of the system parameter a, memristive initial conditions u(0) and other initial conditions z(0), respectively, are analyzed to investigate the complex dynamic properties of the system.

### 3.1. Symmetric Coexistence Attractors Dependent on Parameter a

**Figure 6:**
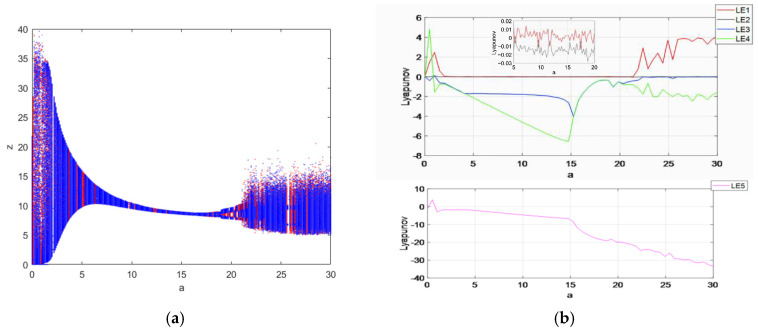
Bifurcation diagram and Lyapunov exponent diagram of chaotic system with a∈ [0,30]: (**a**) bifurcation diagram; (**b**) Lyapunov exponential diagram.

The symmetric coexistence properties of system (3) with parameter a are investigated in this section through means of bifurcation diagrams and a Lyapunov exponential diagram. Let parameter a be a variable control parameter with b=30, c=25, d=10, k=20, m=1 and n=0.01, where the initial condition is (1, 1, 1, 1, 1)(blue) and (−1,(−1, 1,(−1,(−1)(red), the fixed step time is 0.01 s and the total duration is t=200 s. The value of the Lyapunov exponent is calculated by the fourth-order Runge–Kutta method and the wolf algorithm, and the size of the value obtained by the different sampling points taken during the experiment is also different, which also depends on the performance of the computer. By obtaining as many sampling points as possible under the condition of ensuring that the computer can run and improving the accuracy of the value, the number of points taken from the Lyapunov exponent is 600, and the number of points taken from the bifurcation diagram is also 600; the obtained image is shown in Figure 6. The bifurcation diagram of the system state variables z within the parameter a∈ [0,30] is shown in Figure 6a, and the Lyapunov exponential diagram is shown in Figure 6b. From the figure, it can be seen that the system goes through two different states, changing from periodic to chaotic: when a∈ [0,6], a∈ [7,10], a∈ [12.7,17.2] and a∈ [17.8,30], the system is in a chaotic state; when a∈ [6,7], a∈ [10,12.7] and a∈ [17.2,17.8], the system is in a periodic state.

**Figure 7:**
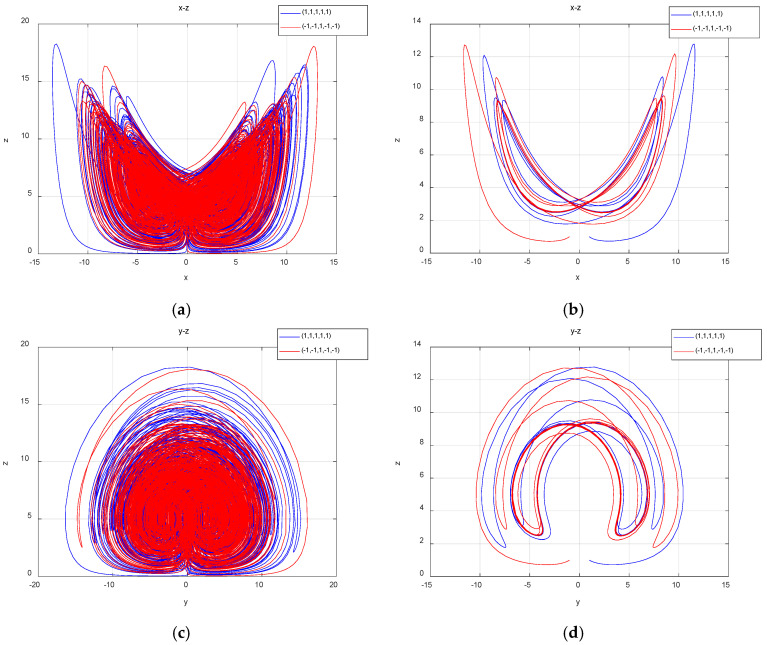
The projections of chaotic attractors and periodic attractors on different planes with initial value symmetry: (**a**) when a=25, the projection of the attractors on the plane x-z is a chaotic system; (**b**) when a=12.7, the projection of the attractors on the plane x-z is periodic; (**c**) when a=25, the projection of the attractors on the plane y-z is a chaotic system; (**d**) when a=12.7, the projection of the attractors on the plane x-z is periodic.

The coexistence attractors with two different initial values were simulated with parameter a of 25 and 12.7; the coexistence attractors of period 4 and chaos were obtained. The chaotic attractors are shown in Figure 7a, the periodic attractors are shown in Figure 7b, the chaotic attractors are shown in Figure 7c and the periodic attractors are shown in Figure 7d.

### 3.2. Analysis of Symmetric Super-Multistability Dependent on Memristive Initial Conditions u(0)

**Figure 8:**
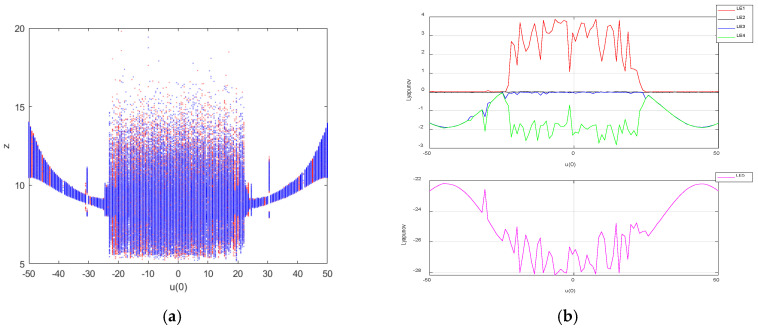
Bifurcation diagram and Lyapunov exponent diagram of chaotic system with u(0)∈ [−50,50]: (**a**) bifurcation diagram; (**b**) Lyapunov exponential diagram.

Keeping the values of the system parameters as a=25, b=30, c=25, d=10, k=20, m=1 and n=0.01, the initial conditions of the system were set to be $X_{1}$ = (1,1,1,1,u(0)) and $X_{2}$ = (−1,−1,1,−1,u(0)), where u(0) is the initial state of the memristor and was set as the bifurcation parameter. The Lyapunov exponent diagram was sampled at 600 points and the bifurcation diagram at 600 points, and the coexistence bifurcation diagrams arising from the initial conditions $X_{1}$ (blue) and $X_{2}$ (red) when the initial value of the memristor u(0) was varied in the interval [−50,50] are shown in Figure 8a, and the Lyapunov exponent diagram is shown in Figure 8b.

When u(0)∈ [−23.7,−22.7], u(0)∈ [26.7,27.7], u(0)∈ [34.8,35.8] and u(0)∈ [42.9,43.9], the system was in a periodic state; when u(0)∈ [−50,−23.7], u(0)∈ [−22.7,26.7], u(0)∈ [27.7,34.8], u(0)∈ [35.8,42.9] and u(0)∈ [43.9,50], the system was in a chaotic state, sometimes in a hyperchaotic state. The analysis shows that the system can exhibit the super-multistability property of the coexistence of an infinite number of attractors under both sets of initial conditions, which is a special nonlinear dynamical phenomenon.

**Figure 9:**
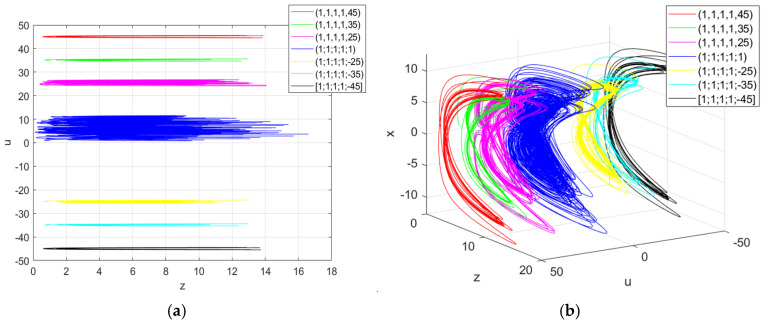
Infinite homogeneous attractors: (**a**) the projection of the attractors on the plane z-u; (**b**) the projection of the attractors on the plane x-z-u.

In order to further reveal this unique super-multistability characteristic, when the initial u(0) of the memristor equals −45, −35, −25, 1, 25, 35 and 45 respectively, the projection plots of the coexisting attractors in different spaces or planes are shown in Figure 9. In addition, when more initial states are selected, the system can exhibit the super-multistability phenomenon, in which an infinite number of attractors coexist.

**Figure 10:**
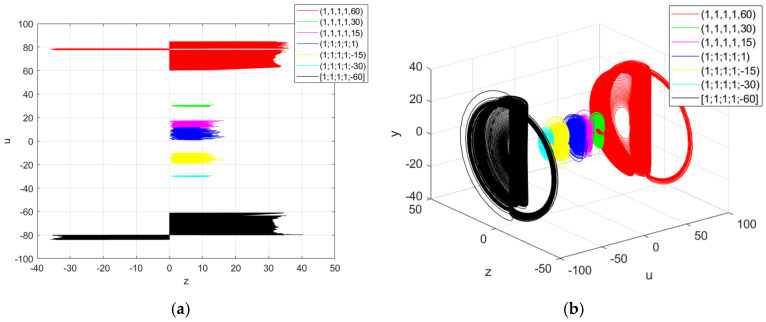
Infinite number of heterogeneous attractors: (**a**) the projection of the attractors on the plane z-u; (**b**) the projection of the attractors on the plane y-z-u.

The parameters were set as above, and the initial values were set as (1,1,1,1,u(0)); when u(0) was taken as 60, 30, 15, 1,(−15,(−30 and –60 respectively, the system generated two-vortex and four-vortex chaotic attractors, as shown in Figure 10. This corresponds to the red, green, pink, blue, yellow, cyan and black chaotic attractors, respectively. It can be observed that these chaotic attractors showed a linear distribution along the u-axis, which is consistent with the results of the previous analysis of the system bifurcation diagram. Through the above analysis, it can be found that the system can produce chaotic attractors with different structures, so the system has heterogeneous multistability, there are infinitely many coexisting attractors and the system behaves as a super-multistability system.

### 3.3. Super-Multistability Analysis Dependent on the Other Initial Value z(0)

**Figure 11:**
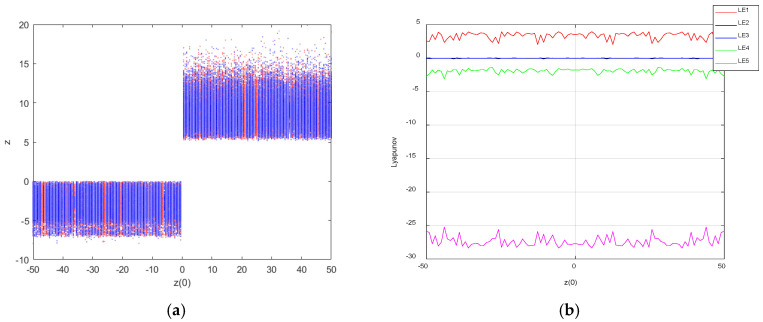
Bifurcation diagram and Lyapunov exponent spectrum of chaotic system with z(0)∈ [−50,50]: (**a**) bifurcation diagram; (**b**) Lyapunov exponential diagram.

Let parameter z(0) be a variable control parameter; when a=25, b=30, c=25, d=10, k=20, m=1 and n=0.01, the initial condition was $X_{1}$ = (1,1,z(0),1,1) and $X_{2}$ = (−1,−1,z(0),−1,−1). The Lyapunov exponent spectrum was sampled at 500 points and the bifurcation diagram at 500 points, and the coexistence bifurcation diagram was drawn for the initial condition z(0)∈ [−50,50]; the Lyapunov exponent spectrum is shown in Figure 11. Infinitely many chaotic states are exhibited on the bifurcation diagram in the range of z(0)∈ [−50,50]. It is further conjectured that system (3) relying on the initial condition z(0) may also generate an infinite number of hidden coexisting attractors; it has hidden super-multistability.

**Figure 12:**
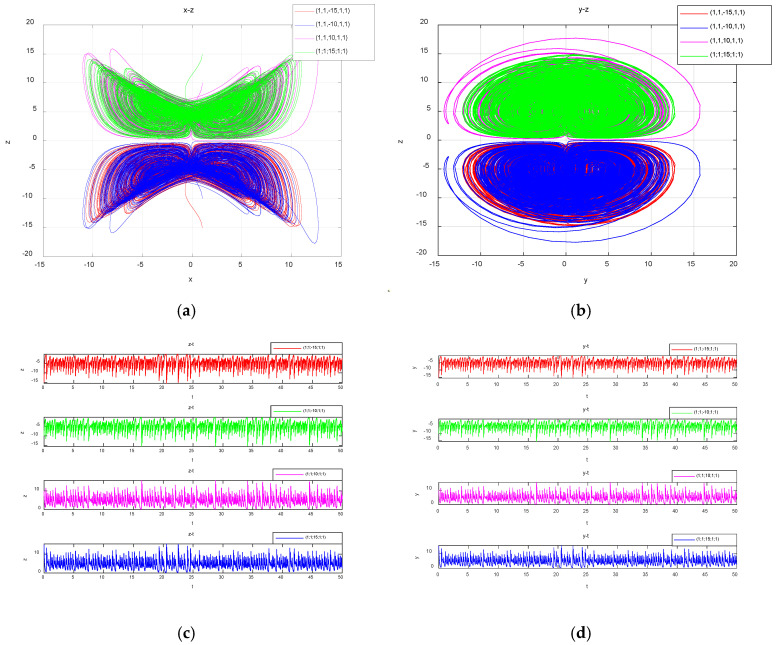
Projections of the attractors on different planes and the time series of z and y in symmetrical coordinates with z(0): (**a**) the projection of the attractors on the plane z-w; (**b**) the projection of the attractors on the plane y-z; (**c**) the time series of z; (**d**) the time series of y.

Taking z(0)= −15, −10, 10 and 15, the corresponding attractors are represented by four colors, red, blue, pink and green respectively; the obtained double-vortex chaotic coexisting attractors and time-domain waveforms in symmetric coordinates are shown in Figure 12.

### 3.4. Effect of the Initial Value of the Memristive u(0) on the Transient Attractors

Transient chaos is a dynamic process in which a system is chaotic for a period of time but evolves into another continuous chaotic or periodic state over time [39,40,41]. The system behaves chaotically for a shorter period of time and enters a periodic behavior after a period of time. The transient chaos and transfer phenomena of the system (3) dependent on the amnesic initial conditions are discussed below.

**Figure 13:**
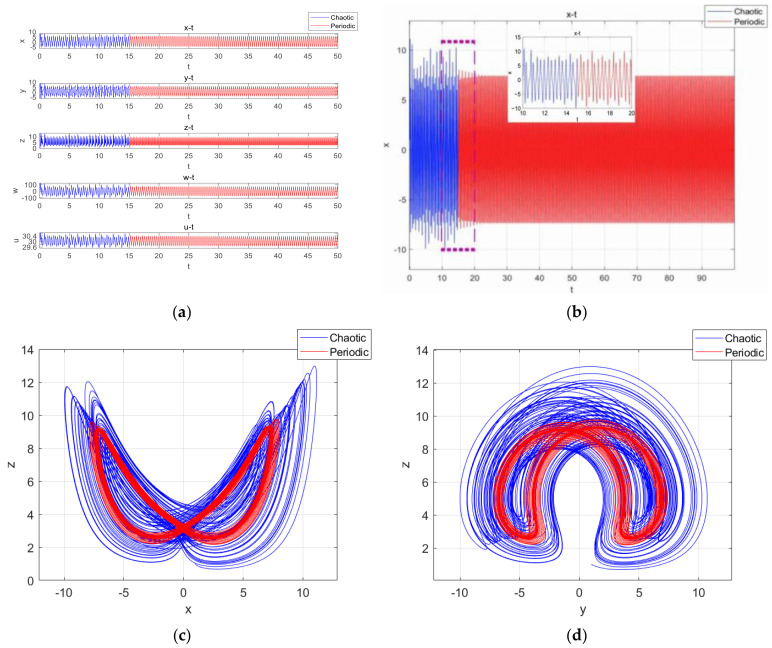
With u(0)=30, transient time-domain waveforms and attractors: (**a**) the time series of x, y, z, w and u; (**b**) the time series of x; (**c**) the projection of the attractors on the plane x-z; (**d**) the projection of the attractors on the plane y-z.

Fixing the parameters as a=25, b=30, c=25, d=10, k=20, m=1 and n=0.01, with the initial condition being (1,1,1,1,u(0)), the simulation time was set to [0,50], and the step size was 0.01. With u(0)=30, the time series of x, y, z, w and u are shown in Figure 13a. Among them, the timing diagram of variable x is shown in Figure 13b, with the simulation time being 0–100. When t=15 s, the time-domain waveforms of the system were transformed from disorganized to regular and orderly, so the system underwent a state transfer at this time. The x-z phase diagram of the motion trajectories in t∈ (0,15] and t∈ (15,50] is shown in Figure 13c, and the y-z phase diagram of the motion trajectories in t∈ (0,15] and t∈ (15,50] is shown in Figure 13d.

Obviously, the system had attractors in the short time before t=15 s and a periodic limit cycle in the longer time after t=15 s. This confirms that the system had transient chaos under the corresponding parameters and initial conditions, and the state transfer behavior occurred near t=15 s, where the blue color represents chaos and the red color represents the cycle.

**Figure 14:**
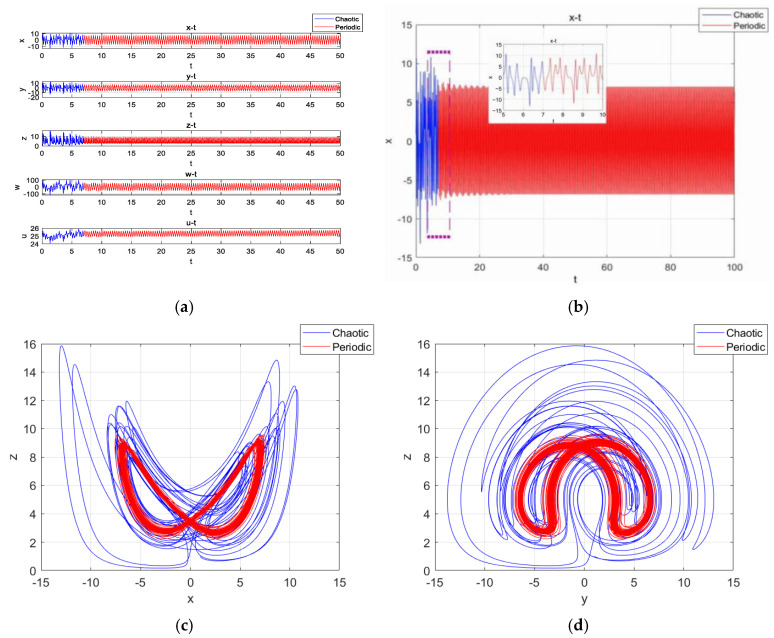
With u(0)=25, transient time-domain waveforms and attractors: (**a**) the time series of x, y, z, w and u; (**b**) the time series of x; (**c**) the projection of the attractors on the plane x-z; (**d**) the projection of the attractors on the plane y-z.

Similarly, when u(0)=25, the system underwent transfer behavior near t=7 s, exhibiting a chaotic state at t∈ (0,7] and a periodic state at t∈ (7,50]. The timing diagrams of the variables x, y, z, w and u are shown in Figure 14a. The timing diagram of variable x-t is shown in Figure 14b. The x-z phase diagram and the y-z phase diagram of the attractors are shown in Figure 14c,d. The simulation results were similar to the previous case, but there was a significant shortening of the existence of transient chaos.

### 3.5. Complexity Analysis

The complexity of a chaotic system is often used to assess the stochastic properties of chaotic sequences. Higher complexity values indicate more random-like sequences, which are particularly valuable for security engineering applications. Currently, there are many algorithms for calculating the complexity of chaotic systems, most of which are based on the Kolmogorov complexity and Shannon entropy. In this paper, the SE algorithm based on the Fourier transform and the C0 algorithm based on the FFT transform are used. The spectral entropy algorithm mainly relies on the Fourier transform, which analyzes the energy distribution in the Fourier transform domain and combines it with the concept of Shannon’s entropy to calculate the spectral entropy value; the core of the C0 algorithm lies in the decomposition of the sequence into regular and irregular parts, and it focuses on the measurement of the proportion of the irregular part of the sequence, which leads to the corresponding results. The specific C0 complexity calculation process is shown as follows.

(1) The first step is to remove the irregular parts of the time series; the mean of the time series is shown in Equation (13).

(13) $$G_{N}=\frac{1}{N}\sum_{k=0}^{N-1}{\left|X(k)\right|}^{2}$$

The parameter r is set to retain the spectrum that exceeds a multiple of the mean value r, and the portion less than or equal to a multiple of the mean value r is set to zero.

(14) $$\tilde{X}(k)=\left\{\begin{array}{l} X(k),{\left|X(k)\right|}^{2}>\xi G_{N}\\ 0,{\left|X(k)\right|}^{2}\leq \xi G_{N} \end{array}\right.$$

(2) In the second step, $\tilde{X}(k)$ is Fourier-inverse-transformed, which is used to obtain Equation (15).

(15) $$\tilde{x}(n)=\frac{1}{N}\sum_{k=0}^{N-1}\tilde{X}(k)e^{j\frac{2\pi }{N}nk}=\frac{1}{N}\sum_{k=0}^{N-1}\tilde{X}(k)W_{N}^{-nk}$$

where n = 0, 1,…, N−1.

(3) The third step is to calculate the C0 complexity, and the formula for the C0 complexity is shown in Equation (16).

(16) $$C_{0}(r,N)=\sum_{n=0}^{N-1}{\left|x(n)-\tilde{x}(n)\right|}^{2}/\sum_{n=0}^{N-1}{\left|x(n)\right|}^{2}.$$

**Figure 15:**
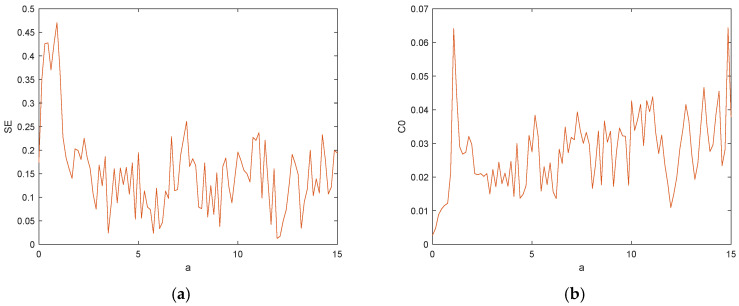
Structural complexity diagram with a: (**a**) the SE complexity of a∈ [0,15]; (**b**) the C0 complexity of a∈ [0,15].

The system complexity measurement is of great significance for the analysis of chaotic system dynamics, and extensive studies have shown that the higher the complexity of chaotic systems, the more suitable they are for secure communication [42]. The SE complexity reflects the disordered state of the Fourier transform domain, and the flatter the spectrum is, the larger its SE value is, which indicates that the complexity of the time series is higher [43]. With the system parameters set as a=25, b=30, c=25, d=10, k=20, m=1 and n=0.01, the initial condition (1, 1, 1, 1, 1) was selected, and a varied in the range of [0,15]; the complexities of SE and C0 of the calculation system are shown in Figure 15.

The trend corresponds to the Lyapunov exponential diagram and the bifurcation diagram in Figure 6. When the system was in a cycle, the corresponding values of SE and C0 were small, and the system complexity was low at this time. When the system was in a chaotic state, the corresponding SE and C0 values were larger, and the system complexity was higher. It can be seen that the system complexity was consistent with the trend of the Lyapunov exponential diagram and bifurcation diagram.

**Figure 16:**
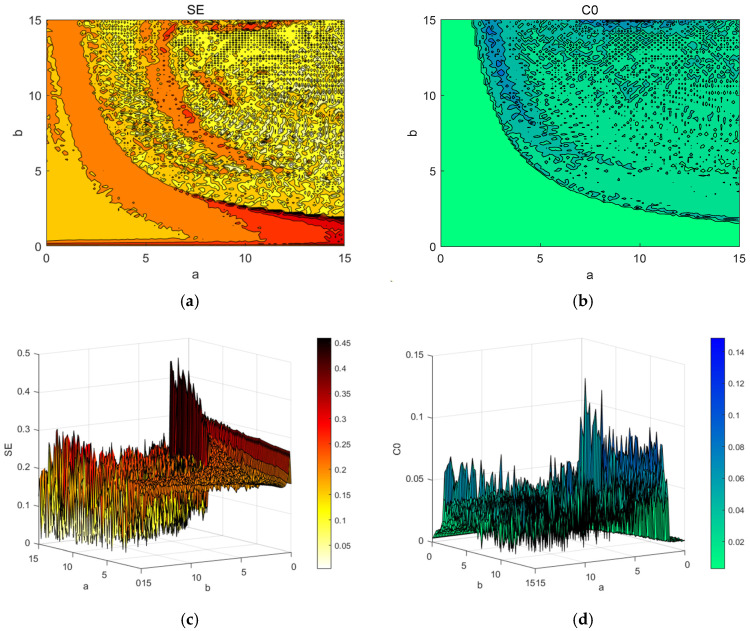
Spectrograms of complexity: (**a**) the SE complexity of a; (**b**) the C0 complexity of a; (**c**) spectrogram of three-dimensional SE complexity with a and b; (**d**) spectrogram of three-dimensional C0 complexity with a and b.

In practical applications, spectrograms of the SE and C0 complexity can provide a basis for better parameter selection. The two-dimensional planar complexity spectrograms and three-dimensional complexity spectrograms of SE and C0 for the two parameters a and b are plotted in Figure 16. The darker the color is, the higher the complexity. In the field of secure communication, a higher complexity indicates greater randomness and a greater difficulty of sequence recovery. Therefore, in practical applications, the complexity of the chaotic system should be increased to ensure that the system has good anti-interference properties.

## 4. Circuit Simulation

Chaotic circuits are essentially chaotic systems, and electronic circuits are usually utilized to generate chaotic signals and to demonstrate the physical existence of a chaotic system. Therefore, circuit implementation has become a necessary step for chaotic systems to move from theory to practical engineering applications. Here, a simple analog circuit is designed to verify the dynamical behavior of the new memristor super-chaotic system. The circuit includes circuit components such as linear capacitors, linear resistors, operational amplifiers and analog multipliers. Among them, the operational amplifier was chosen to be an LM2924N device with an operating voltage of ±15 V, and the analog multiplier was an AD633 device. A time scale transformation was performed, such that $\tau =\tau_{0}t$, where $\tau_{0}=100$. The parameters were selected as follows: a=25, b=30, c=25, d=10, k=20, m=1 and n=0.01. System (3) can be represented as follows:

(17) $$\left\{\begin{array}{l} \overset{•}{x}=-2500x+3000y+100w\\ \overset{•}{y}=2500x-100xz^{2}+100\\ \overset{•}{z}=100xyz-1000z\\ \overset{•}{w}=2000y+20u^{2}y-100w\\ \overset{•}{u}=100y \end{array}\right..$$

Applying Kirchhoff’s circuit law, the following equation of state is obtained:

(18) $$\left\{\begin{array}{l} \overset{•}{x}=-\frac{1}{R_{1}C_{1}}x+\frac{1}{R_{2}C_{1}}y+\frac{1}{R_{3}C_{1}}w\\ \overset{•}{y}=\frac{1}{R_{6}C_{2}}x-\frac{1}{R_{7}C_{2}}xz^{2}+\frac{1}{R_{8}C_{2}}V_{1}\\ \overset{•}{z}=\frac{1}{R_{11}C_{3}}xyz-\frac{1}{R_{12}C_{3}}z\\ \overset{•}{w}=\frac{1}{R_{15}C_{4}}y+\frac{1}{R_{16}C_{4}}u^{2}y-\frac{1}{R_{17}C_{4}}w\\ \overset{•}{u}=\frac{1}{R_{20}C_{5}}y \end{array}\right..$$

**Figure 17:**
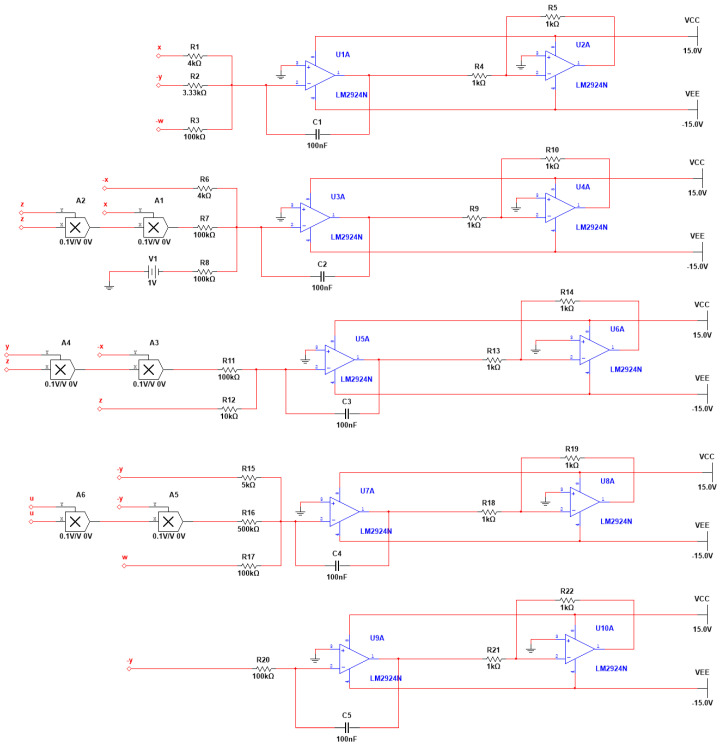
Schematic diagram of state variables x, y, z, w and u circuits.

**Figure 18:**
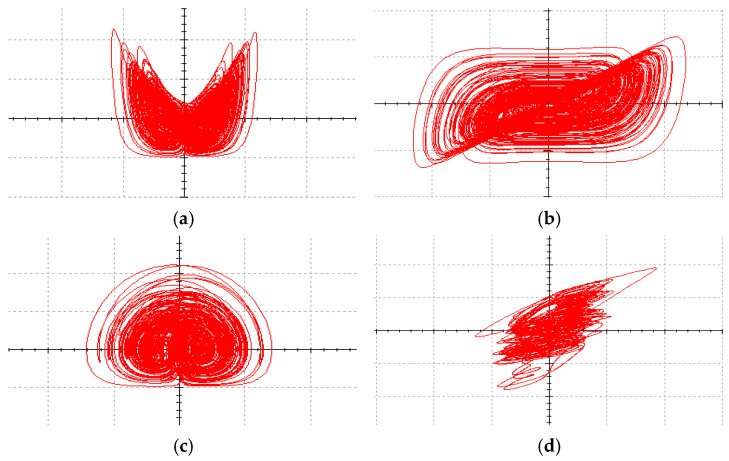
Projections of the attractors on different planes: (**a**) the projection of the attractors on the plane x-z; (**b**) the projection of the attractors on the plane x-y; (**c**) the projection of the attractors on the plane y-z; (**d**) the projection of the attractors on the plane x-w.

Let the capacitance $C_{1}=C_{2}=C_{3}=C_{4}=C_{5}=100\;nF$, and with the voltage set as $V_{1}=1\;V$, the parameters of the circuit components can be obtained as follows: $R_{1}=4\;k\Omega$, $R_{2}=3.33\;k\Omega$, $R_{6}=4\;k\Omega$, $R_{12}=10\;k\Omega$, $R_{15}=5\;k\Omega$, $R_{16}=500\;k\Omega$, $R_{3}=R_{7}=R_{8}=100\;k\Omega$, $R_{11}=R_{17}=R_{20}=100\;k\Omega$, $R_{4}=R_{5}=R_{9}=R_{10}=R_{13}=R_{14}=R_{18}=R_{19}=R_{21}=R_{22}=1\;k\Omega$. The circuit schematic of system (3) was obtained by the corresponding derivation shown in Figure 17. Then, the Multisim software 14.3 was used to simulate the circuit, and the simulation results are shown in Figure 18. From the figure, it can be seen that the projection results of the hyperchaotic attractors in the different planes obtained by the Multisim circuit simulation were consistent with the numerical simulation results shown in Figure 1. The existence and realizability of the memristive hyperchaotic system are thus verified.

## 5. Synchronization Control of Chaotic System

### 5.1. Chaotic System Synchronization

Considering that system (3) has parameter uncertainties, an adaptive control method was employed to design the controller for the synchronized control of system (3). Let system (3) be a driven system; the formula is defined as follows:

(19) $$\left\{\begin{array}{l} \overset{•}{x_{1}}=-ax_{1}+by_{1}+w_{1}\\ \overset{•}{y_{1}}=cx_{1}-{x_{1}z_{1}}^{2}+1\\ {\overset{•}{z}}_{1}=x_{1}y_{1}z_{1}-dz_{1}\\ \overset{•}{w_{1}}=20(1+0.01u_{1}^{2})y_{1}-w_{1}\\ {\overset{•}{u}}_{1}=y_{1} \end{array}\right.$$

where $x_{1}$, $y_{1}$, $z_{1}$, $w_{1}$ and $u_{1}$ are system variables; and a, b, c and d are unknown parameters.

The response system with the control term is calculated as follows:

(20) $$\left\{\begin{array}{l} \overset{•}{x_{2}}=-ax_{2}+by_{2}+w_{2}+v_{1}\\ \overset{•}{y_{2}}=cx_{2}-{x_{2}z_{2}}^{2}+1+v_{2}\\ {\overset{•}{z}}_{2}=x_{2}y_{2}z_{2}-dz_{2}+v_{3}\\ \overset{•}{w_{2}}=20(1+0.01u_{2}^{2})y_{2}-w_{2}+v_{4}\\ {\overset{•}{u}}_{1}=y_{2}+v_{5} \end{array}\right.$$

where $x_{2}$, $y_{2}$, $z_{2}$, $w_{2}$ and $u_{2}$ are the system variables, and $v_{1}$, $v_{2}$, $v_{3}$, $v_{4}$ and $v_{5}$ are the adaptive synchronization controllers for the system.

The synchronization error between the drive system and the response system is defined as follows:

(21) $$\left\{\begin{array}{l} e_{1}=x_{2}-x_{1}\\ e_{2}=y_{2}-y_{1}\\ e_{3}=z_{2}-z_{1}\\ e_{4}=w_{2}-w_{1}\\ e_{5}=u_{2}-u_{1} \end{array}\right.$$

Substituting Equations (19) and (20) into Equation (21), the error dynamics are obtained as follows:

(22) $$\left\{\begin{array}{l} {\overset{•}{e}}_{1}=-ae_{1}+be_{2}+e_{4}+v_{1}\\ {\overset{•}{e}}_{2}=ce_{1}-x_{2}z_{2}^{2}+x_{1}z_{1}^{2}+v_{2}\\ {\overset{•}{e}}_{3}=-x_{1}y_{1}z_{1}+x_{2}y_{2}z_{2}-de_{3}+v_{3}\\ {\overset{•}{e}}_{4}=20e_{2}+0.2u_{2}^{2}y_{2}-0.2u_{1}^{2}y_{1}+v_{4}\\ {\overset{•}{e}}_{5}=e_{2}+v_{5} \end{array}\right.$$

The designed adaptive controller is shown as follows.

(23) $$\left\{\begin{array}{l} v_{1}=\hat{a}e_{1}-\hat{b}e_{2}-e_{4}-k_{1}e_{1}\\ v_{2}=-x_{1}z_{1}^{2}+x_{2}z_{2}^{2}-\hat{c}e_{1}-k_{2}e_{2}\\ v_{3}=x_{1}y_{1}z_{1}-x_{2}y_{2}z_{2}+\hat{d}e_{3}-k_{3}e_{3}\\ v_{4}=-20e_{2}-0.2u_{2}^{2}y_{2}+0.2u_{1}^{2}y_{1}-k_{4}e_{4}\\ v_{5}=-e_{2}-k_{5}e_{5} \end{array}\right.$$

where $k_{1}$*,* $k_{2}$*,* $k_{3}$*,* $k_{4}$ and $k_{5}$ are the positive gain constants used to control the synchronization speed of the chaotic system, and $\hat{a}$*,* $\hat{b}$*,* $\hat{c}$ and $\hat{d}$ are the parameter estimates of the unknown parameters a*,* b*,* c and d, respectively.

Substituting Equation (23) into Equation (22), the synchronization error dynamics equation, Equation (24), is obtained as follows:

(24) $$\left\{\begin{array}{l} {\overset{•}{e}}_{1}=-(a-\hat{a})e_{1}+(b-\hat{b})e_{2}-k_{1}e_{1}\\ {\overset{•}{e}}_{2}=(c-\hat{c})e_{1}-k_{2}e_{2}\\ {\overset{•}{e}}_{3}=-(d-\hat{d})e_{3}-k_{3}e_{3}\\ {\overset{•}{e}}_{4}=-k_{4}e_{4}\\ {\overset{•}{e}}_{5}=-k_{5}e_{5} \end{array}\right.$$

The parameter estimation error dynamics are given by Equation (25).

(25) $$\left\{\begin{array}{l} e_{a}=a-\hat{a}\\ e_{b}=b-\hat{b}\\ e_{c}=c-\hat{c}\\ e_{d}=d-\hat{d} \end{array}\right.$$

The parameter estimation error dynamics are derived in Equation (26).

(26) $$\left\{\begin{array}{l} {\overset{•}{e}}_{a}=-\overset{•}{\hat{a}}\\ {\overset{•}{e}}_{b}=-\overset{•}{\hat{b}}\\ {\overset{•}{e}}_{c}=-\overset{•}{\hat{c}}\\ {\overset{•}{e}}_{d}=-\overset{•}{\hat{d}} \end{array}\right.$$

Substituting Equation (25) into Equation (24) yields the following equation:

(27) $$\left\{\begin{array}{l} {\overset{•}{e}}_{1}=-e_{a}e_{1}+e_{b}e_{2}-k_{1}e_{1}\\ {\overset{•}{e}}_{2}=e_{c}e_{1}-k_{2}e_{2}\\ {\overset{•}{e}}_{3}=-e_{d}e_{3}-k_{3}e_{3}\\ {\overset{•}{e}}_{4}=-k_{4}e_{4}\\ {\overset{•}{e}}_{5}=-k_{5}e_{5} \end{array}\right.$$

It follows from the Lyapunov stability theory that the Lyapunov function is defined as follows:

(28) $$V=\frac{1}{2}(e_{1}^{2}+e_{2}^{2}+e_{3}^{2}+e_{4}^{2}+e_{5}^{2}+e_{a}^{2}+e_{b}^{2}+e_{c}^{2}+e_{d}^{2})$$

Derivation of this yields the following:

(29) $$\begin{array}{ll} \overset{•}{V}= & -k_{1}e_{1}^{2}-k_{2}e_{2}^{2}-k_{3}e_{3}^{2}-k_{4}e_{4}^{2}-k_{5}e_{5}^{2}+e_{a}(-e_{1}^{2}-\hat{a})+e_{b}(e_{1}e_{2}-\hat{b})\\ & +e_{c}(e_{1}e_{2}-\hat{c})+e_{d}(-e_{3}^{2}-\hat{d}) \end{array}.$$

In Equation (29), the following parameters are obtained:

(30) $$\left\{\begin{array}{l} -e_{1}^{2}-\hat{a}=0\\ e_{1}e_{2}-\hat{b}=0\\ e_{1}e_{2}-\hat{c}=0\\ -e_{3}^{2}-\hat{d}=0 \end{array}\right..$$

In conclusion, the following parameters are obtained:

(31) $$\left\{\begin{array}{l} \hat{a}=-e_{1}^{2}\\ \hat{b}=e_{1}e_{2}\\ \hat{c}=e_{1}e_{2}\\ \hat{d}=-e_{3}^{2} \end{array}\right..$$

Substituting Equation (31) into Equation (29) yields the following equation:

(32) $$\overset{•}{V}=-k_{1}e_{1}^{2}-k_{2}e_{2}^{2}-k_{3}e_{3}^{2}-k_{4}e_{4}^{2}-k_{5}e_{5}^{2}\leq 0.$$

This leads to $\overset{•}{V}\leq 0$, which shows that V is negative and semidefinite. According to the Lyapunov stability theory, the system is uniformly stable at equilibrium if V is positive and definite and V is negative and semi-definite. It shows that the drive system (19) and the response system (20) are eventually synchronized.

**Figure 19:**
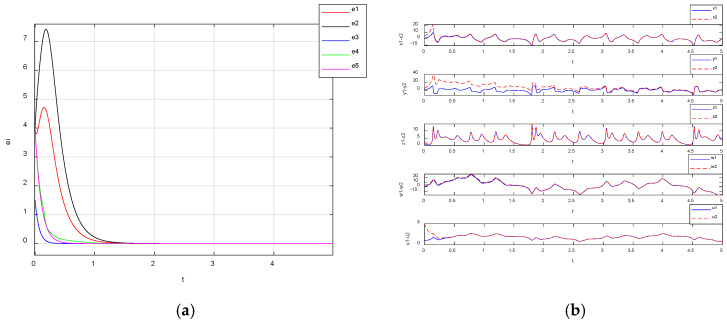
Nonlinear feedback synchronization error plot and waveform convergence curves: (**a**) nonlinear feedback synchronization error plot; (**b**) waveform convergence curves.

By setting the parameters of system (3) in the chaotic state with the parameters as a=25, b=30, c=25, d=10 and the gain as K=10 and setting the initial value of the driving system as (1, 1, 1, 1, 1) and the response system as (5, 5, 2.5, 5, 5), the synchronization error curves of system (3) were obtained through simulation, as shown in Figure 19a. From Figure 19a, it is observed that the synchronization errors e1, e2, e3, e4 and e5 were synchronized at about t=1.5 s, and they were asymptotically stabilized to zero in a short time. Meanwhile, the synchronized waveforms of all the variables when this hyperchaotic system reached synchronization are shown in Figure 19b, and it can be seen from Figure 19b that the waveforms x1-x2, y1-y2, z1-z2, w1-w2 and u1-u2 of the driving system and the responding system converged to the same level within a short period of time, which demonstrates that the two systems achieved synchronization.

Compared with the adaptive sliding-mode synchronization method used in the literature [44], the time required to reach synchronization was about 20 s, while the adaptive synchronization control method used in this paper could reach synchronization within 1.5 s, demonstrating that the adaptive synchronization control method proposed in this paper is more effective in achieving the synchronization of chaotic systems.

### 5.2. Application of Chaotic System Synchronization in Secure Communication

With the rapid development of computer technology, information security has received more and more attention. Secure communication based on chaotic synchronization has developed into an important branch of information security research, so this paper uses chaotic masking secure communication technology to realize the secure transmission of signals on the basis of realizing the synchronization of a memristive chaotic system. The chaotic masking secure communication is designed to superimpose the signal generated by the chaotic system on the information signal to be encrypted to form a modulated signal and then transmit it through the information channel. Then, the receiver is used to demodulate the modulated mixed signal through the output signal generated by the chaotic system after synchronization with the transmitter; the original useful signal is restored, and the function of the synchronous control unit is used to realize the synchronization between the driving system and the response system, which is the key to chaotic secure communication.

**Figure 20:**
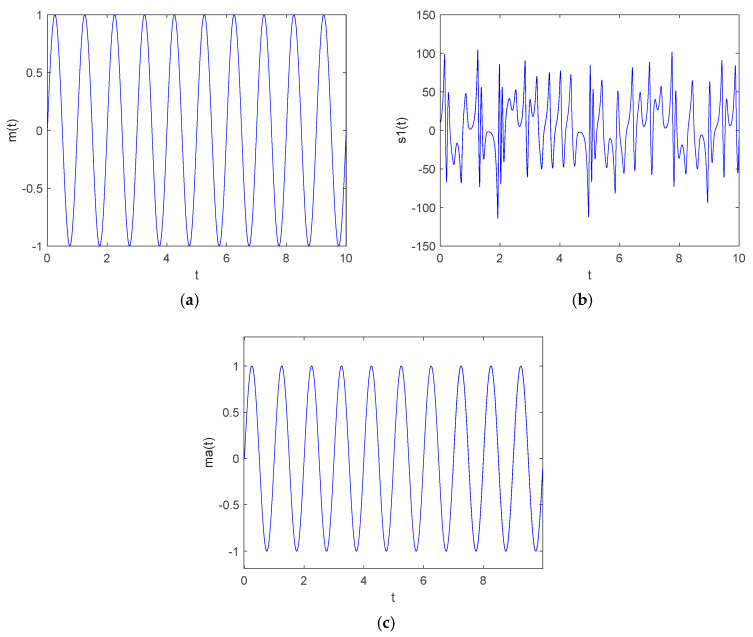
Encryption and decryption results of chaotic masking secure communication signals: (**a**) original signal; (**b**) encrypted signal; (**c**) decrypted signal.

Firstly, the state variable $y_{1}(t)$ was selected as the carrier signal, and the demodulation signal was $y_{2}(t)$. The original signal to be encrypted was m(t)=sin(t); then, the encrypted signal was $s_{1}(t)=m(t)+y_{1}(t)$, and the signal $s_{1}(t)$ was sent to the receiver output signal $s_{2}(t)$ through the channel in the ideal state $s_{1}(t)=s_{2}(t)$. Then, the signal $s_{2}(t)$ was used to subtract the demodulated chaotic signal $y_{2}(t)$ generated by the synchronized response system implemented by the synchronization controller to obtain the decrypted information signal $m_{a}$. The system parameters remained unchanged, the initial value of the drive system was set to (1, 1, 1, 1, 1), and the initial value of the response system was (5, 5, 2.5, 5, 5). According to the principle of chaotic masking secure communication encryption, the simulation results of the signal encryption and decryption were obtained by MATLAB numerical simulation, as shown in Figure 20.

## 6. Conclusions

In this paper, a five-dimensional memristor chaotic system was innovatively constructed using a magnetically controlled memristor. Firstly, compared with the previous chaotic systems, the new system improved the complexity, and it exhibited strong sensitivity to parameter variations and initial conditions, so the multistability of the system was analyzed by means of a Lyapunov exponential diagram, bifurcation diagrams and phase portraits. Upon varying the system parameter a, the initial state of the memristive component and other initial conditions, the experimental results show that the system exhibited a rich behavior of periodic chaotic alternation associated with the parameters, symmetric attractors, transient chaotic behaviors, homogeneous multistability, heterogeneous multistability and super-multistability phenomena. Secondly, two offsets, g and h, were introduced into the linear state variables of the system as the displacement controllers of the system in the z-axis and w-axis directions, respectively, and the effects of the offsets on the dynamical behavior of the system were analyzed. The complexity of the system was analyzed by using the SE algorithm and the C0 algorithm. The analysis results show that the values of the SE and the C0 algorithms increased with the increase in the complexity of the system, and the color of the three-dimensional spectrograms became darker. In addition, simulation experiments of the five-dimensional chaotic system were carried out using Multisim to verify the feasibility of the system design. Finally, this paper adopted adaptive synchronization and designed a corresponding adaptive synchronization controller. By adjusting the positive gain constant K=10 and setting the initial values of the driving and response systems to (1, 1, 1, 1, 1) and (5, 5, 2.5, 5, 5), respectively, synchronization between the driving system and the response system was achieved. Based on chaotic synchronization, the chaotic masking method was used to encrypt and decrypt a sinusoidal signal. In the future, this signal encryption scheme can continue to be optimized, and hardware implementation based on this encryption scheme can be realized, which can provide stronger support and expansion space for the development of communication security, data protection and privacy assurance.

## Figures and Tables

**Table 1:** The state of the chaotic system corresponding to the 0–1 test when the parameter a was varied.

a	0–1 Test Results	Chaotic System States
a=12.7	Regular and boundary	Cycle 4
a=14.2	Regular and boundary	Cycle 4
a=16.5	Regular and boundary	Cycle 7
a=25	Irregular and indefinitely growing	chaotic

## Data Availability

Data included in the article.
